# Laser-Induced Deposition of Plasmonic Ag and Pt Nanoparticles, and Periodic Arrays

**DOI:** 10.3390/ma14010010

**Published:** 2020-12-22

**Authors:** Daria V. Mamonova, Anna A. Vasileva, Yuri V. Petrov, Denis V. Danilov, Ilya E. Kolesnikov, Alexey A. Kalinichev, Julien Bachmann, Alina A. Manshina

**Affiliations:** 1 Institute of Chemistry, Saint-Petersburg State University, 26 Universitetskii Prospect, Saint-Petersburg 198504, Russia; magwi@mail.ru (D.V.M.); anvsilv@gmail.com (A.A.V.); julien.bachmann@fau.de (J.B.); 2 Department of Physics, Saint-Petersburg State University, Ulyanovskaya 3, Saint-Petersburg 198504, Russia; y.petrov@spbu.ru; 3 Interdisciplinary Resource Center for Nanotechnology, Research Park, Saint-Petersburg State University, Ulyanovskaya 1, Saint-Petersburg 198504, Russia; danilov1denis@gmail.com; 4 Centre for Optical and Laser Materials Research, Research Park, Saint-Petersburg State University, Ulyanovskaya 5, Saint-Petersburg 198504, Russia; ilya.kolesnikov@spbu.ru (I.E.K.); kalinichev.alex@gmail.com (A.A.K.); 5 Department of Chemistry and Pharmacy, Friedrich–Alexander University of Erlangen—Nürnberg, IZNF, Cauerst. 3, 91058 Erlangen, Germany

**Keywords:** laser-induced deposition, noble metal NPs, plasmon resonance, nano-grating structures

## Abstract

Surfaces functionalized with metal nanoparticles (NPs) are of great interest due to their wide potential applications in sensing, biomedicine, nanophotonics, etc. However, the precisely controllable decoration with plasmonic nanoparticles requires sophisticated techniques that are often multistep and complex. Here, we present a laser-induced deposition (LID) approach allowing for single-step surface decoration with NPs of controllable composition, morphology, and spatial distribution. The formation of Ag, Pt, and mixed Ag-Pt nanoparticles on a substrate surface was successfully demonstrated as a result of the LID process from commercially available precursors. The deposited nanoparticles were characterized with SEM, TEM, EDX, X-ray diffraction, and UV-VIS absorption spectroscopy, which confirmed the formation of crystalline nanoparticles of Pt (3–5 nm) and Ag (ca. 100 nm) with plasmonic properties. The advantageous features of the LID process allow us to demonstrate the spatially selective deposition of plasmonic NPs in a laser interference pattern, and thereby, the formation of periodic arrays of Ag NPs forming diffraction grating

## 1. Introduction

Laser fabrication methods provide specific physicochemical conditions in the zone of processing. As a result, they open efficient and highly controlled ways of nanomaterials synthesis with specific functionality. Metal nanoparticles (NPs) are a particular class of objects that are of interest due to their plasmonic properties and that can be synthesized with laser technologies. Among the most widespread techniques of metal NPs synthesis, are pulsed laser ablation/deposition in vacuum, gas, or liquid phases [1,2,3], as well as direct synthesis under intense laser beam focused in a solutions of metal salts [4,5]. These methods demonstrate high processing efficiency and a wide list of metal NPs have been synthesized with controlled parameters. However, their application for the surface functionalization with NPs has been difficult.

Other promising laser technologies rely on the creation of surfaces decorated/functionalized with metal NPs. One of them is the colorization of metal surfaces due to formation of plasmonic NPs by means of metal surface treatment with pulsed laser ablation [6]. Laser-induced forward transfer (LIFT) achieves the spatially controlled formation of a donor material (that can be a metal film as well) on a receiving substrate [7]. As a hybrid method of surface functionalization with metal NPs, preliminary surface activation with femtosecond laser beam, followed by laser induced photoreduction, can be mentioned [8].

All the listed methods are based on the use of highly intense pulsed laser radiation that imposes some restrictions to their applicability and the list of substrates that can be decorated with metal nanostructures. Among the methods combining both the ability of surface decoration with metal NPs and the use of a continuum-wave laser irradiation is the group of laser-induced depositions (LID). The LID approach is based on laser irradiation of the substrate–solution interface with a laser beam. As a result of laser-initiated chemical processes, metal structures are formed in the laser-affected area of the substrate. As a liquid phase, one can use electrolyte solutions traditional for chemical metallization [9,10,11], or solutions of metal salts with some reducing agents [12,13,14,15,16], or solutions of organometallic complexes [17,18]. The main difference between these approaches is the nature of the laser-induced process—the redox process initiated by laser heating (first case) or photochemical effects in the second and third variants. LID processes based on laser heating (often referred as laser-induced chemical liquid phase deposition of metals—LCLD) typically use laser irradiation of visible range with rather high power (hundreds of mW). Though LCLD allows precipitation of the functional, electrically conducting metal structures [19], its main limitation is the high-temperature process of reduction of metal ions and the side effects of local decomposition of precipitate and substrate. These side effects lead to inhomogeneous deposition of the metal phase, and as a result, rather high specific resistance of the deposits [20].

The photo-induced LID process uses laser irradiation of low intensity to photo-initiate the decomposition of an organometallic complex (that is the main component of the solution) dissolved in an appropriate solvent. As the next step, the formation of nanostructures from the precursor components takes place on the surface of the substrate. The main feature of the LID process is the spectral selectivity of the process (the laser wavelength should coincide with absorption band of the precursor), allowing one to use the low-intensity continuous-wave laser irradiation. The LID process proved to be very flexible, efficient, and well-controlled. It is characterized by good correlation of the chemical composition of the precursor and the deposited structures that is a consequence of the mild conditions of laser irradiation. The main competitive advantage of LID over other laser-based methods is its ability to synthesize NPs directly on any given/preselected surface area of the substrate [21,22]. It allows ‘on-site’ functionalization of various types of surfaces with NPs active in catalysis and electrocatalysis, surface-enhanced Raman spectroscopy (SERS), plasmon-enhanced fluorescence, energy conversion, solar cell technologies, etc. [17,18,23,24].

To date, the LID decoration of different surfaces has been successfully demonstrated—such as 2D substrates (microscope cover glasses and Si wafers both pristine and covered by indium tin oxide film) and 3D substrates (nanowires, capillaries, and porous anodic alumina membranes) [22,25,26].

As a main constraint of LID, one can consider the limited number of organometallic complexes demonstrated so far as LID precursor. In most cases, home-made supramolecular complexes were used that were heterometallic transition–metal polynuclear complexes. This kind of complex is of high interest for the LID process as they have precisely defined central bimetallic cluster core, allowing synthesis of bimetallic nanoparticles in the form of alloys. However, the preliminary synthesis procedure of home-made organometallic complexes can be considered as an obstacle to a widespread application of LID.

Here, we present the successful laser-induced deposition of monometallic Ag and Pt nanoparticles, and mixed Ag/Pt samples from commercially available precursors—organic salts, organometallic complexes without reducing agents adding to the solutions. This opens wide perspectives to LID and makes it generally available. We demonstrate precise control over the morphology and composition of the deposits by means of routine variation of the LID experimental parameters. As another LID advantage, we present spatially selective deposition of plasmonic NPs in a laser interference pattern. It opens the possibility of ‘on-site’ creation of nano-grating structures formed of periodically distributed arrays of NPs. The sensitivity of the deposits’ morphology to the parameters of the LID process is demonstration of its flexibility and potency for creation of fine structures for tailoring light-matter interaction and sensing technologies development.

## 2. Materials and Methods

### 2.1. Materials and Reagents

Analytical grade solvents (dichlorethane, dichlormethane, isopropanol, ethanol, methanol, acetonitrile, hexane) were purchased from Reachem (Reachem, Moscow, Russia), and used after standard purification procedures [27]. In addition, for solutions preparation, double-distilled water was used. Cover glass and quartz slips with 0.15 mm thickness and 5 mm × 5 mm size (Levenhuk G100 cover slips; model 5932-020-00288679-2012) (Tampa, FL, USA) were used as substrates for LID. As a source of metal, we used two silver-containing precursors and two platinum-containing precursors: silver benzoate hydrate (silver salt C_6_H_5_COOAg, containing of Ag 47.1 wt%), silver bis(trifluoromethylsulfonyl)azanide (silver salt, (CF_3_SO_2_)_2_NAg containing of Ag 27.6 wt%), ethenyl-[ethenyl(dimethyl)silyl]oxy-dimethylsilane platinum in vinyl terminated polydimethylsiloxane 0.1 M solution (platinum complex C_8_H_18_OPtSi_2_, containing of Pt 3 wt%), (1Z,5Z)-cycloocta-1,5-diene dichloroplatinum (platinum salt (C_8_H_12_)Cl_2_Pt, containing of Pt 51.5 wt%). Precursors were purchased from Alfa Aesar and used as received. The molecular structures of these substances are presented in Figure S1 Supporting Information.

### 2.2. Laser-Induced Deposition

The compositions of the precursors’ solutions for the laser-induced deposition are presented in Table 1. Preparation of the solutions was carried out with ultrasonic treatment for 5 min and subsequent centrifugation at 12,000 rpm for 3 min in a Sigma 2–16P (Sigplma Laborzentrifugen, Osterode am Harz, Germany). Because of low solubility of (C_8_H_12_)Cl_2_Pt in C_6_H_14_ and H_2_O and low miscibility of vinyl terminated polydimethylsiloxane with H_2_O, these solutions were not used for further LID experiments.

All the prepared solutions were considered for laser-induced deposition of monometallic (Pt and Ag) structures; the binary Pt-Ag system was deposited from mixed acetonitrile solution of C_6_H_5_COOAg (7.5 mM) and C_8_H_18_OPtSi_2_ in vinyl terminated polydimethylsiloxane (3.9 mM). The absorption spectra of all the studied solutions are presented in Supplementary Materials (Figure S2). The laser wavelength for the LID process was chosen in accordance with characteristic absorption bands of the studied precursors. That is why a solid-state, continuous wave, single frequency, deep-UV laser system Coherent MBD266 (wavelength 266 nm, power 60 mW, unfocused laser beam, laser spot diameter 2 mm) was used as a radiation source for LID realization. The laser parameters were kept the same for all LID experiments.

**Figure 1:**
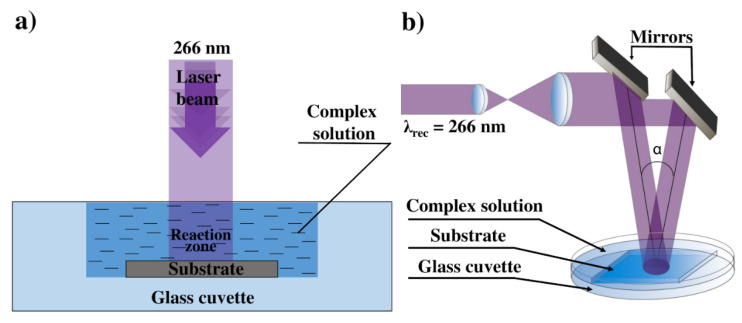
(**a**) Reaction system of LID; (**b**) Experimental setup for synthesis of periodic silver nanoparticle structures using holographic recording.

The scheme of the LID process is presented in Figure 1a. Unfocused laser beam with diameter ca. 2 mm was directed to the substrate–solution interface through the solution. The volume of the cuvette was 80 µL, thickness of the solution 1 mm. LID was carried out in a stationary regime—no shift of laser beam relative to the substrate. Laser irradiation time was 40 min in all the experiments. In the case of platinum complexes, LID process was performed both on quartz substrates and cover glass slips, deposition from silver complexes was performed on cover glass slips. After the LID process, the substrates were washed with isopropanol and dried at ambient conditions.

To investigate the effect of laser intensity on the LID process, the formation of deposits in the laser interference pattern was studied. A regular two-beam scheme was used for the interference pattern construction [28]. The scheme of the experimental setup is shown in Figure 1b. Two parallel laser beams of equal intensities were produced by dividing the input laser beam that was expanded and collimated before. These two coherent beams were directed at an angle *α* to each other to form an interference pattern. The period *d* of the interference pattern was determined by the formula $2dsin\left(\alpha /2\right)=\lambda_{rec}$, where *λ_rec_* is a laser wavelength. The LID process was performed for two interference patterns—with 375 and 750 lines/mm. The LID process in the interference pattern was carried out similarly to the LID in a single laser beam. As a deposition solution in the interference pattern, we used 0.3 mM silver benzoate hydrate C_6_H_5_COOAg in methanol. The LID process in the interference pattern was carried out for laser power 45 and 200 mW and a range of irradiation time from 2 to 20 min, the irradiated area was ca. 0.5 cm^2^.

### 2.3. Samples Characterization

The absorption spectra of solutions were recorded with SHIMADZU UV-2550 over the spectral range 200–500 nm with 1 nm step, medium scan speed, and 1 nm slit width. The absorption spectra of NPs deposited on cover glass or quartz slip were recorded with a Lambda 1050 (Perkin Elmer, Waltham, MA, USA) in the range of 300–550 nm (for silver NPs) and 200–550 nm (for platinum NPs) with an integrating Ulbricht sphere. The substrate with NPs was placed in the sphere center. The morphology and composition of obtained samples were investigated by scanning electron microscopy (SEM) and energy-dispersive X-ray spectroscopy (EDX) with a scanning electron microscope Zeiss Merlin (Oberkochen, Germany) with field emission cathode, GEMINI electron-optics column, oil-free vacuum system, using variable pressure charge compensation mode with local nitrogen injection. No conductive coating was used. Crystallinity and composition of the samples were investigated by transmission electron microscopy (TEM) with Zeiss Libra 200. For TEM investigation, the samples were sonicated in isopropanol and deposited onto the carbon-coated TEM grid. The crystal structure was also studied by X-ray diffractometer Bruker “D8 DISCOVER” (Billerica, MA, USA), using Cu Kα X-ray line. Atomic force microscopy (AFM) (NT-MDT, Moscow, Russia) analysis of deposits obtained under interference pattern was performed with NTEGRA-Prima setup in tapping mode in areas of 2 × 60 µm and 14 × 14 µm with 256 points/line.

## 3. Results and Discussion

### 3.1. Characterization of Monometallic Ag, Pt Nanostructures Obtained by LID

**Figure 2:**
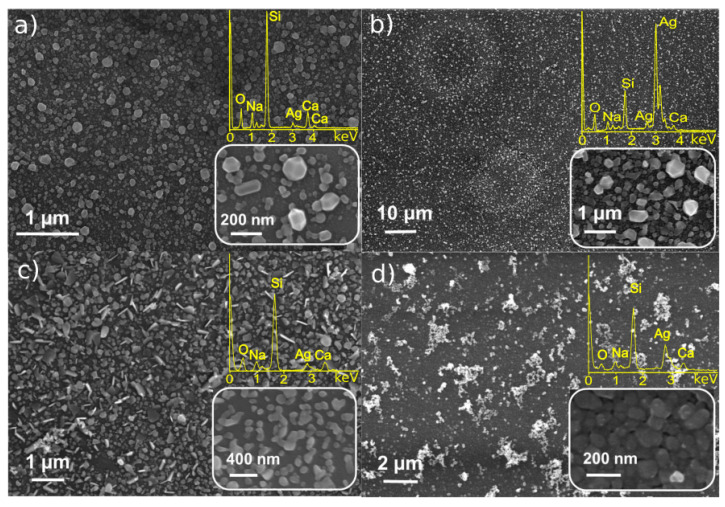
SEM/EDX analysis of Ag particles on cover glass synthesized from (**a**) C_6_H_5_COOAg in methanol (0.4 mM), (**b**) C_6_H_5_COOAg in acetonitrile (1.3 mM), (**c**) (CF_3_SO_2_)_2_NAg in ethanol (3.3 mM), (**d**) (CF_3_SO_2_)_2_NAg in isopropanol (2.8 mM).

**Figure 3:**
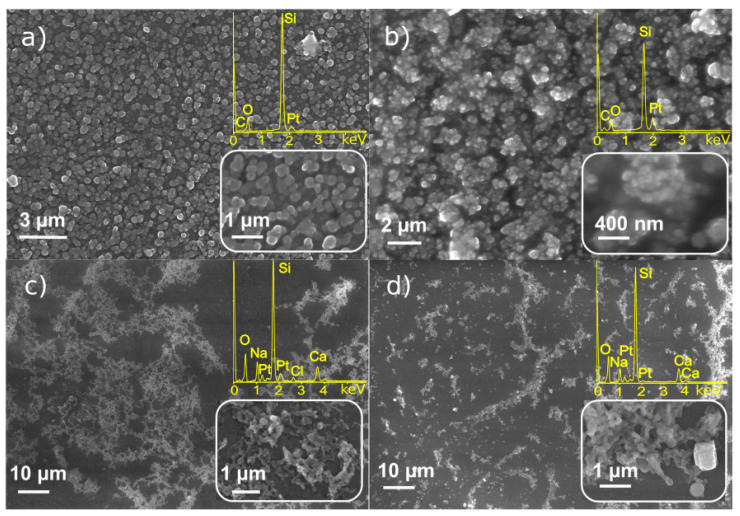
SEM/EDX analysis of particles synthesized from (**a**) C_8_H_18_OPtSi_2_ in vinyl terminated polydimethylsiloxane (0.1 M), (**b**) C_8_H_18_OPtSi_2_ in hexane C_6_H_14_ (3.9 mM), (**c**) (C_8_H_12_)Cl_2_Pt in dichloromethane CH_2_Cl_2_ (5.3 mM), (**d**) (C_8_H_12_)Cl_2_Pt in dichlorethane C_2_H_4_Cl_2_ (1.3 mM).

Laser-induced deposition for studied Pt and Ag precursors was found to be successful for all the solutions listed in Table 1. The result of the LID process was the formation of nanostructures in the laser-affected area of the substrate. The density and homogeneity of the nanostructure distribution on the substrate surface exhibited some spatial variation depending on the precursor and the solvent. The most typical deposits are presented in Figure 2 and Figure 3.

Figure 2 demonstrates the SEM images of the structures obtained by the LID procedure from silver precursors (C_6_H_5_COOAg and (CF_3_SO_2_)_2_NAg). The LID process results in formation of nanoparticles that are homogeneously distributed on the substrate’s surface. The NPs are characterized by a faceted morphology in the case of C_6_H_5_COOAg precursor with rather wide size distribution: 50–200 nm for the methanol solvent and 100–500 nm for acetonitrile. The larger size of NPs from C_6_H_5_COOAg solution in acetonitrile is most likely determined by higher concentration of C_6_H_5_COOAg in acetonitrile than in methanol, as all other deposition parameters were kept identical.

The LID from (CF_3_SO_2_)_2_NAg precursor also results in homogeneously distributed NPs; in the case of (CF_3_SO_2_)_2_NAg in isopropanol, the layer of NPs is thicker. The NPs do not reveal faceted morphology—(CF_3_SO_2_)_2_NAg solution in ethanol results in formation of platelets, while (CF_3_SO_2_)_2_NAg in isopropanol demonstrates aggregated NPs; the average size is about 150 nm for both solvents. EDX analysis testifies that all the deposited NPs consist of silver; the observed O, Na, Si, Ca are signals from the substrates (cover slips), no other impurities were found.

The SEM images of nanostructures obtained from platinum precursors are presented in Figure 3. The deposition from C_8_H_18_OPtSi_2_ precursor demonstrates formation of NPs with highly homogeneous distribution onto the substrate’s quartz surface (Figure 3a,b). Dilution with hexane of “native” precursor solution in vinyl terminated polydimethylsiloxane results in the formation of less continuous coatings consisting of agglomerated NPs.

Figure 3c,d presents SEM images of nanostructures deposited from (C_8_H_12_)Cl_2_Pt precursor. Generally, the deposits do not form homogeneous coating as distinct from the C_8_H_18_OPtSi_2_ complex (Figure 3a,b). The morphology of structures synthesized from solutions of (C_8_H_12_)Cl_2_Pt in dichloroethane and dichloromethane is similar; the size of particles is close, but in case of dichloromethane, we see a higher coverage. EDX analysis testifies the presence of Pt in all the deposited samples, however in the case of C_8_H_18_OPtSi_2_ precursor, one can see C and O signals that probably originate from the precursor complex. O, Na, Si, Ca peaks in Figure 3c,d are due to the signal from the microscope cover slip substrate.

Thus, SEM and EDX analysis of the structures obtained by LID technique demonstrates that the most homogeneous and regular structures consisted of Pt and Ag nanoparticles can be obtained correspondingly from C_8_H_18_OPtSi_2_ and C_6_H_5_COOAg precursors. Variations of the solvent have pronounced effects, and enable the experimentalist to adjust the morphology of the deposit. For the further experiments on LID synthesis of the binary Pt-Ag system, the mixed solution of C_8_H_18_OPtSi_2_ and C_6_H_5_COOAg precursors in acetonitrile was chosen.

### 3.2. Characterization of Binary Pt-Ag System Obtained by LID

**Figure 4:**
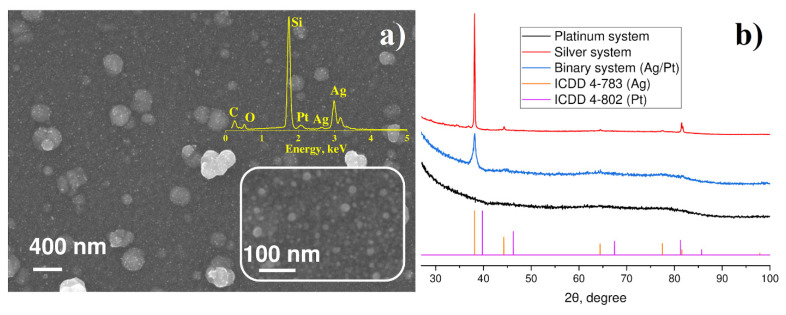
(**a**) SEM/EDX analysis of the of particles synthesized from mixed Pt-Ag precursors, (**b**) X-ray diffraction of particles on quartz substrates for platinum system C_8_H_18_OPtSi_2_; silver system (C_6_H_5_COOAg in acetonitrile); binary system (C_8_H_18_OPtSi_2_+ C_6_H_5_COOAg in acetonitrile); ICDD PDF-2 for platinum (4-802 card) and silver (4-783 card).

LID from the mixed solution of the C_8_H_18_OPtSi_2_ and C_6_H_5_COOAg precursors in acetonitrile resulted in the formation of a continuous coating on the substrate surface (Figure 4a). One can see a rather homogeneous distribution of 20–30 nm NPs with several larger particles of 200–400 nm that are aggregates of small NPs. In spite of similarity in the morphologies of monometallic (Ag, Pt) and binary Pt-Ag NPs, the characterization of the latter requires more detailed analysis and additional techniques to uncover the composition and structure of the deposited phase.

Figure 4b presents experimental XRD patterns of nanoparticles obtained from C_6_H_5_COOAg and C_8_H_18_OPtSi_2_ complexes, respectively, their mixture, and the PDF-2 database cards for silver and platinum. One can see that Ag NPs demonstrated pronounced diffraction peaks, which coincide well with the silver card ICDD 4-783 for both monometallic and binary systems. NPs obtained from C_8_H_18_OPtSi_2_ complexes did not show any trace of diffraction peaks and displayed only diffuse halo. This fact can be explained by formation of amorphous platinum NPs or small size of Pt crystallites. To determine the correct reason, further investigation of synthesized samples was carried out by means of electron microscopy and microanalysis.

**Figure 5:**
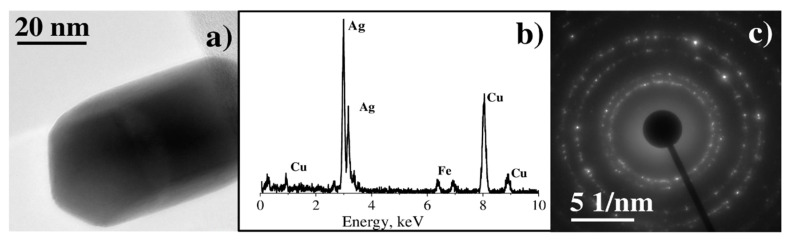
(**a**) Bright-field (BF) TEM image of large single crystal, (**b**) EDX spectrum, (**c**) electron diffraction pattern.

TEM analysis of deposits from C_6_H_5_COOAg precursor demonstrate the formation of rather large crystalline particles, with diameters on the order of 100–200 nm (Figure 5a), that consist of silver in accordance with EDX microanalysis (Figure 5b). Copper and iron peaks are attributed to the sample grid and specimen holder. The electron diffraction pattern (Figure 5c) consists of several rings, as typical for randomly oriented crystals. Measuring the diameters of these rings, we have obtained the interplanar spacings of 2.40 Å, 2.08 Å, 1.46 Å, and 1.22 Å, which are identical within experimental uncertainty to literature values for the spacings in face-centered cubic lattice of silver (2.36 Å, 2.04 Å, 1.44 Å, and 1.18 Å) [29]. Thus, both electron diffraction and EDX microanalysis (Figure 5b,c) unambiguously show that these particles consist of silver only.

**Figure 6:**
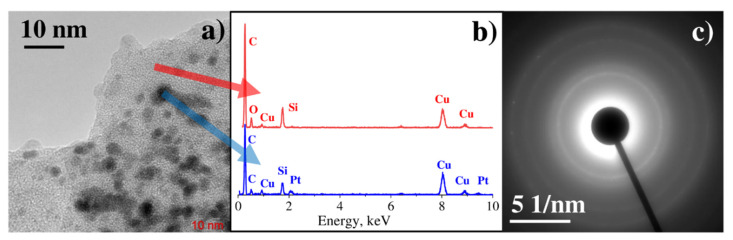
(**a**) BF TEM image, (**b**) EDX spectrum from single nanoparticle and spectrum from the matrix, (**c**) electron diffraction pattern.

As one can observe from the BF TEM image (Figure 6a), the sample deposited from the C_8_H_18_OPtSi_2_ precursor consists of crystalline nanoparticles in an amorphous matrix. EDX analysis of single particle demonstrates several peaks in the spectrum, which correspond to the C Kα line, O Kα line, Pt Mα line, and Si Kα line (Figure 6b, blue spectrum). The spectrum measured from an amorphous matrix (Figure 6b, red spectrum) includes C Kα line, O Kα line, and Si Kα line. Thus, it can be concluded that the sample consists of Pt nanoparticles (ca. 1–3 nm in diameter) incorporated into an amorphous matrix, consisting of carbon, silicon, and oxygen. The origin of the amorphous matrix is the decomposition of ligand in the precursor’s complex C_8_H_18_OPtSi_2_. Several rings with diameters corresponding to the interplanar spacings of 2.29 Å, 1.98 Å, 1.41 Å, and 1.20 Å are observed in electron diffraction (Figure 6c), which can be attributed to crystalline Pt (2.27 Å, 1.96 Å, 1.39 Å, 1.18 Å) [30].

**Figure 7:**
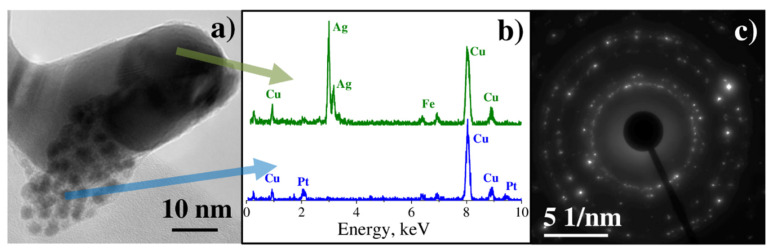
(**a**) Bright-field (BF) TEM image of sample 1-2.1, (**b**) X-ray spectrum from small nanoparticles and spectrum from large nanoparticles, (**c**) electron diffraction pattern.

TEM investigation of the sample deposited from the mixture of C_6_H_5_COOAg and C_8_H_18_OPtSi_2_ complexes shows that it consists of two types of nanoparticles: small ones with the size of several nanometers, and large ones with the size of hundreds of nanometers (Figure 7a).

The EDX spectrum measured from small nanoparticles (Figure 7b, blue spectrum) exhibits an X-ray peak at 2.05 keV that corresponds to the Pt Mα line. An EDX spectrum measured from large nanoparticles (Figure 7b, green spectrum) shows a prominent peak at 2.98 keV that corresponds to the Ag Lα line. Electron diffraction consists of several rings and some bright reflexes (Figure 7c). The reflexes correspond to the interplanar spacings: 2.40 Å, 2.08 Å, 1.46 Å, and 1.22 Å, typical for crystalline silver, whereas rings correspond to 2.29 Å, 1.98 Å, 1.41 Å, and 1.20 Å, typical for crystalline platinum.

Therefore, the detailed analysis performed with SEM, TEM, EDX, and X-ray diffraction techniques unambiguously revealed the formation of ca. 100 nm crystalline nanoparticles of silver as a result of the LID process from C_6_H_5_COOAg and (CF_3_SO_2_)_2_NAg precursors, while Pt deposits are characterized by smaller size 1–5 nm. It should be noted that despite the absence of specific X-ray reflexes corresponding to Pt crystals in the diffraction patterns of Pt and mixed Pt-Ag samples, TEM electron diffraction gives unambiguous confirmation of crystalline nature of Pt deposits.

It should be noted that the detailed description of the mechanism of formation of Ag and Pt NPs from the solutions of precursors studied here require additional investigation. Nevertheless, keeping in mind the resonance absorption of laser radiation by precursor molecules and the absence of reducing agent as a component of the solution, one can conclude that laser excitation of the precursor molecules is followed by their decomposition/transformation and intramolecular redox processes resulting in formation of metal phase. In the case of the C_8_H_18_OPtSi_2_ precursor, we found ligand incorporation into the structure of deposit along with Pt clusters. LID from the binary Ag-Pt solutions demonstrated independent processes of formation of Ag and Pt phases resulting in precipitation of mixture of NPs.

### 3.3. LID-Obtained Structures for Optical Application

The nanostructures deposited in the current study can be considered as prospective material for application in a range of fields, for example, surface-enhanced Raman spectroscopy (SERS) or ultrasensitive optical sensors based on plasmonic enhancement of electromagnetic fields. Plasmonic properties of metal nanoparticles reveal in UV-VIS absorption spectra as characteristic bands, which positions depend on metal nature [31], size, and shape of NPs [32], and refractive index of the media [33]. In case of multimetallic NPs, the characteristic absorption bands are sensitive to the nature of metal phase (alloy or mechanical mixture) [34,35]. Thus, absorption spectroscopy can be considered as a sensitive tool for characterization of both physicochemical properties of NPs and their functionality.

**Figure 8:**
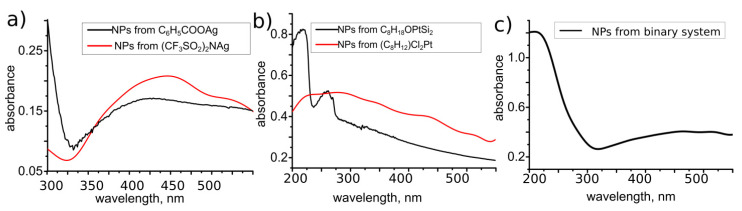
Absorption spectra of nanoparticles (NPs) deposited from (**a**) C_6_H_5_COOAg in methanol and (CF_3_SO_2_)_2_NAg in isopropanol; (**b**) C_8_H_18_OPtSi_2_ in vinyl terminated polydimethylsiloxane and (C_8_H_12_)Cl_2_Pt in dichloromethane; (**c**) mixture of C_6_H_5_COOAg and C_8_H_18_OPtSi_2_ in acetonitrile.

Figure 8 shows absorption spectra of NPs deposited as a result of the LID process from solutions of C_6_H_5_COOAg, (CF_3_SO_2_)_2_NAg, C_8_H_18_OPtSi_2_, and (C_8_H_12_)Cl_2_Pt precursors. Rather broad bands in range 375–475 nm observed for NPs from Ag-containing precursors (Figure 8a) correspond to the plasmon-related absorption of silver nanostructures and testify to a wide size distribution of NPs. The faceted elongated shape of silver NPs confirmed by SEM can also contribute to the plasmonic spectra width [36].

Absorption spectra of NPs from Pt-containing precursors (Figure 8b) demonstrate more specific features, especially for the solution of C_8_H_18_OPtSi_2_. One can see pronounced peaks at 220 and 260 nm, which are attributed to the intra-band electronic transitions from energy states of (*n* = 5; l = 2) and (*n* = 6; l = 0) to higher energy states of the conduction bands of Pt NPs [37,38]. Pt NPs obtained from (C_8_H_12_)Cl_2_Pt precursor display a wide absorption band centered at about 270 nm. The width of the band may be caused by overlapping of peaks related with faceted shape of NPs and broad sizes distribution. Difference of absorption spectra of Pt NPs deposited from various precursors could be due to size and shape variation.

It is interesting to note that absorption spectra of deposits obtained from the mixed solution of C_8_H_18_OPtSi_2_ and C_6_H_5_COOAg precursors display two separated peaks with maxima 220 and 450 nm. The peaks positions are typical for Pt and Ag nanoparticles, respectively, and their presence confirms the formation of a binary system consisting of a mixture of Pt and Ag NPs. These data coincide well with the described above TEM analysis and absorption spectra for monometallic NPs.

**Figure 9:**
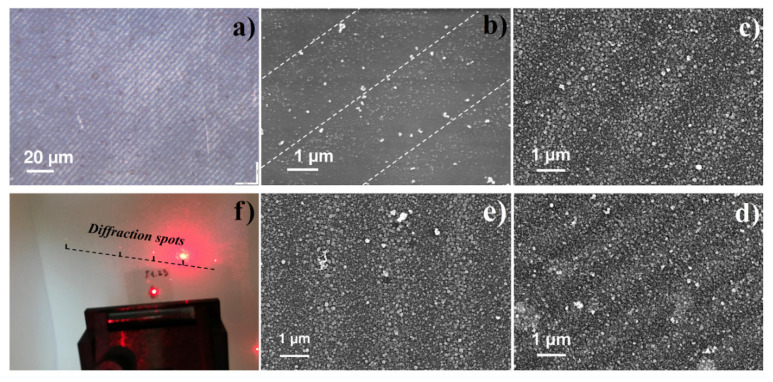
Micrograph and SEM analysis of Ag NPs grating with different synthesis conditions: (**a**,**b**) laser power of 200 mW, irradiation time 2 min, period 375 lines/mm; (**c**) laser power of 45 mW, irradiation time 20 min, period 375 lines/mm; (**d**) laser power of 45 mW, irradiation time 40 min, period 750 lines/mm; (**e**) laser power of 200 mW, irradiation time 10 min, period 375 lines/mm; (**f**) demonstration of diffraction effect on Ag NPs grating deposited under laser power of 200 mW, irradiation time 10 min, period 375 lines/mm.

The peculiarity of the LID presented here is the use of low intensity laser irradiation and laser wavelengths corresponding to the characteristic absorption of the precursor, all together proving the photo-induced nature of NPs’ formation process. In this case, the deposition process can be sensitive to the spatial variation of laser intensity and promising for the controlled NPs distribution on the substrate surface. As a proof of concept, the LID process was realized in an interference pattern for the C_6_H_5_COOAg precursor in methanol. Figure 9 shows an optical micrograph and SEM images of Ag NPs gratings obtained in different synthesis conditions (laser power, deposition time, diffraction period). One can see high sensitivity of the deposits’ morphology on the synthesis parameters. Deposition at high laser power (200 mW) and short laser irradiation time (2 min) generates periodic arrays with and without NPs (Figure 9a,b), but its quality is not high enough for demonstration of diffraction. Decreasing the laser power while keeping short illumination time does not result in formation of a periodic pattern of NPs. Further optimization of deposition parameters results in a full coverage of the substrate surface with NPs, at the same time, the periodicity of the morphology is clearly observed (Figure 9c–e). It is interesting to note that the average NPs’ size for the ‘line’ and ‘gap’ areas is close (ca. 30 nm) but the size distribution is narrower in case of ‘gap’ areas (S3). Figure 9f demonstrates diffraction of the He–Ne laser beam (633 nm, 5 mW) on the sample, presented in Figure 9e.

**Figure 10:**
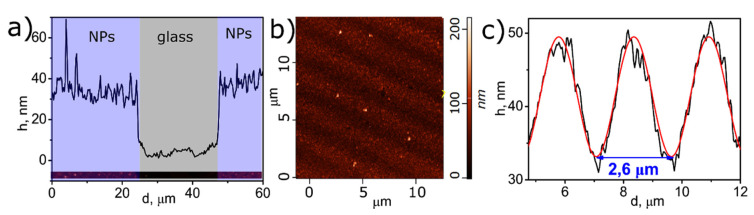
(**a**) AFM image and average profile of LID-synthesized sample; (**b**) AFM image of Ag NPs grating deposited under laser beam with interference pattern 375 lines/mm; (**c**) average profile (black line) and fitting waveform (red line) of synthesized periodic structure.

The morphology of the deposited gratings was studied with AFM analysis. AFM images of the cross-section and surface of the sample (laser power of 45 mW, irradiation time 40 min, interference pattern 375 lines/mm) are given in Figure 10. An average thickness of the sample of ca. 40 nm was obtained as a result of a scan through a scratch line. Figure 10b confirms the formation of a surface relief grating with height (difference between minima and maxima) approximately 25 nm and a period ca 2.6 µm, according to Figure 10c. The results obtained coincide perfectly with the interference pattern period that was 2.67 µm for the sample studied here.

## 4. Conclusions

In this work, we successfully demonstrated the formation of Ag, Pt, and mixed Ag-Pt nanoparticles on the surface of glass and quartz slips as a result of the LID process from solutions of commercially available precursors—C_6_H_5_COOAg, (CF_3_SO_2_)_2_NAg, C_8_H_18_OPtSi_2_, and (C_8_H_12_)Cl_2_Pt. The deposition process was found to be possible from solutions of all the studied precursors in a wide range of solvents. A pronounced effect of solvent on the deposits’ morphology was observed which can be used to deposit NPs with desired properties. The deposited NPs were characterized with SEM, TEM, EDX, X-ray analysis. In the case of deposition from the solutions containing one type of precursors, formation of crystalline platinum NPs with average size 1–5 nm, and silver nanoparticles ca. 100 nm was found. Deposition from the binary Ag-Pt solutions revealed formation of a mixture of monometallic crystalline Ag and Pt NPs that testifies toward independent processes of Ag and Pt phase formation during LID. X-ray analysis revealed good correlation of Ag reflexes with database, while Pt reflexes were not detected. At the same time, electron diffraction patterns of the studied samples demonstrated interplanar spacings typical for crystalline silver and crystalline platinum. The obtained X-ray results show that absence of crystalline features in XRD patterns does not necessary testify amorphous phase, independent investigation with electron diffraction can be more informative towards small crystallites detection.

UV-VIS absorption spectroscopy demonstrated plasmonic absorption of all the studied samples and gave independent confirmation of formation of NPs mixture in the case of the Ag-Pt sample. It should be noted that the mechanism of NPs formation from the solutions of the studied precursors requires further understanding; however, resonance absorption of laser radiation by precursor molecules and the following intramolecular redox processes resulting in formation of metal nano-species can be considered as a general description of the LID process.

The unique feature of the LID procedure presented is the use of low-intensity laser irradiation that provides the photo-induced nature of the deposition process, the absence of the destructive effect of laser radiation on the deposits, and the potential for precise control of the deposits’ spatial distribution. As a proof of concept, the spatially selective deposition of plasmonic NPs in the laser interference pattern was successfully demonstrated. Diffraction gratings with different parameters 375 lines/mm and 750 lines/mm formed of periodically distributed Ag NPs were created and characterized. The obtained results open a wide range of opportunities for the LID process. Firstly, the list of potential precursors can be extended towards commercially available precursors to make LID widely accessible. Secondly, a number of applications can be explored where different kinds of plasmonic effects are utilized (tailoring light, non-invasive optical detection methods, sensing technologies).

## Figures and Tables

**Table 1:** Concentrations (mM) of precursors in solutions used for laser-induced deposition (LID).

Solvent	Precursor			
	C_6_H_5_COOAg	(CF_3_SO_2_)_2_NAg	C_8_H_18_OPtSi_2_	(C_8_H_12_)Cl_2_Pt
C_2_H_4_Cl_2_	4.5	2.6	1.3	1.3
CH_3_OH	0.4	0.8	26.2	0.2
C_6_H_14_	2.2	4.2	3.9	-
C_3_H_8_O	1.1	2.8	13.1	3.0
H_2_O	1.7	0.5	-	-
C_2_H_3_N	1.3	1.3	3.9	2.7
CH_2_Cl_2_	1.3	0.8	2.9	5.3
C_2_H_5_OH	0.9	3.3	3.9	4.8

## Data Availability

Please refer to suggested Data Availability Statements in section “MDPI Research Data Policies” at https://www.mdpi.com/ethics. Studies” of Research Park of Saint Petersburg State University for technical support.

## Supplementary Materials

The following are available online at https://www.mdpi.com/1996-1944/14/1/10/s1, Figure S1: Structures of LID precursors, Figure S2: Absorbance spectra of precursors in different solvents (a) C7H5AgO2; (b) C2AgF6NO4S2; (c) O[Si(CH3)2 CH=CH2]2Pt; (d) C8H12Cl2Pt; Figure S3: (a) Particles size histogram for line area of Ag NPs grating; (b) SEM for line and gap areas of Ag NPs grating, laser power of 200 mW, irradiation time 20 min periods of the interference pattern 3 m (c) Particles size histogram for gap area of Ag NPs grating.
