# Supplementary material for: Laser-Induced Deposition of Plasmonic Ag and Pt Nanoparticles, and Periodic Arrays
Source: Materials (Basel). 2020 Dec 22;14(1):10. doi: 10.3390/ma14010010 (PMC7792966; doi:10.3390/ma14010010)
Supplement: Supplementary file 1 [file materials-14-00010-s001.pdf]

materials

Supplementary

# Laser-Induced Deposition of Plasmonic Ag and Pt Nanoparticles, and Periodic Arrays

**Daria V. Mamonova [1], Anna A. Vasileva [1], Yuri V. Petrov [2], Denis V. Danilov [3], Ilya E. Kolesnikov [4], Alexey A. Kalinichev [4], Julien Bachmann [1,5] and Alina A. Manshina [1,*]**

1. Institute of Chemistry, Saint-Petersburg State University, 26 Universitetskii Prospect, Saint-Petersburg 198504, Russia; magwi@mail.ru (D.V.M.); anvsilv@gmail.com (A.A.V.); julien.bachmann@fau.de (J.B.)
2. Department of Physics, Saint-Petersburg State University, Ulyanovskaya 3, Saint-Petersburg 198504, Russia; y.petrov@spbu.ru
3. Interdisciplinary Resource Center for Nanotechnology, Research Park, Saint-Petersburg State University, Ulyanovskaya 1, Saint-Petersburg 198504, Russia; danilov1denis@gmail.com
4. Centre for Optical and Laser Materials Research, Research Park, Saint-Petersburg State University, Ulyanovskaya 5, Saint-Petersburg 198504, Russia; ilya.kolesnikov@spbu.ru (I.E.K.); kalinichev.alex@gmail.com (A.A.K.)
5. Department of Chemistry and Pharmacy, Friedrich–Alexander University of Erlangen—Nürnberg, IZNF, Cauerst. 3, 91058 Erlangen, Germany

* Correspondence: a.manshina@spbu.ru

**Citation:** Mamonova, D.V.; Vasileva, A.A.; Petrov, Y.V.; Danilov, D.V.; Kolesnikov, I.E.; Kalinichev, A.A.; Bachmann, J.; Manshina, A.A. Laser-Induced Deposition of Plasmonic Ag and Pt Nanoparticles, and Periodic Arrays. *Materials* **2020**, *14*, 10. https://doi.org/10.3390/ma14010010

Received: 30 November 2020
Accepted: 21 December 2020
Published: date

**Publisher's Note:** MDPI stays neutral with regard to jurisdictional claims in published maps and institutional affiliations.

**Copyright:** © 2020 by the authors. Submitted for possible open access publication under the terms and conditions of the Creative Commons Attribution (CC BY) license (http://creativecommons.org/licenses/by/4.0/).

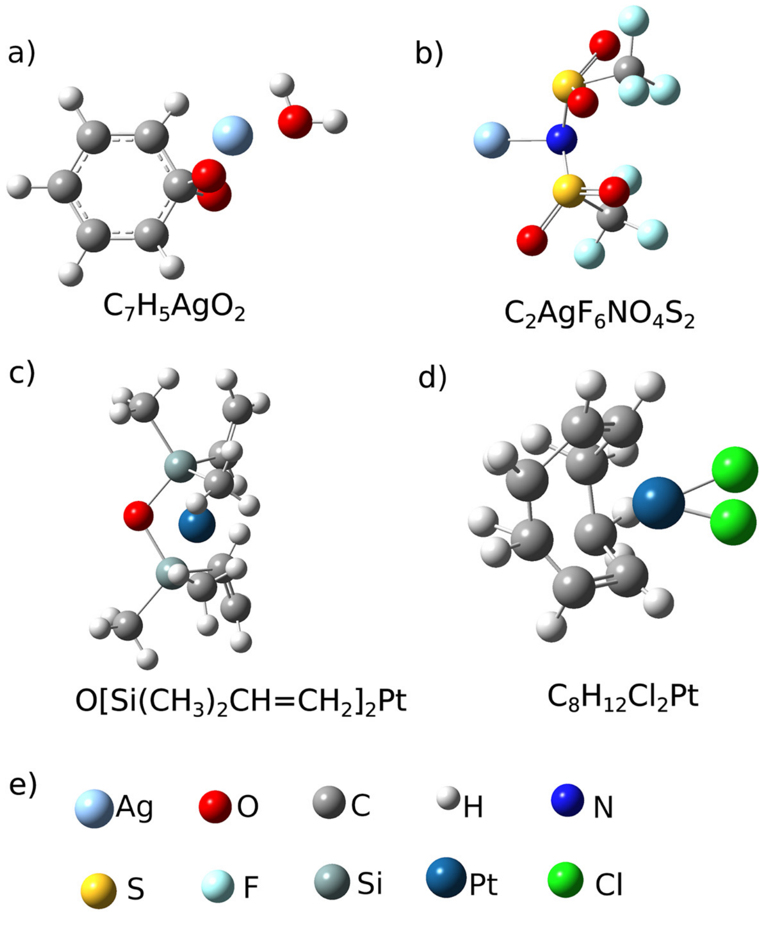


**Figure S1.** Structures of LID precursors.

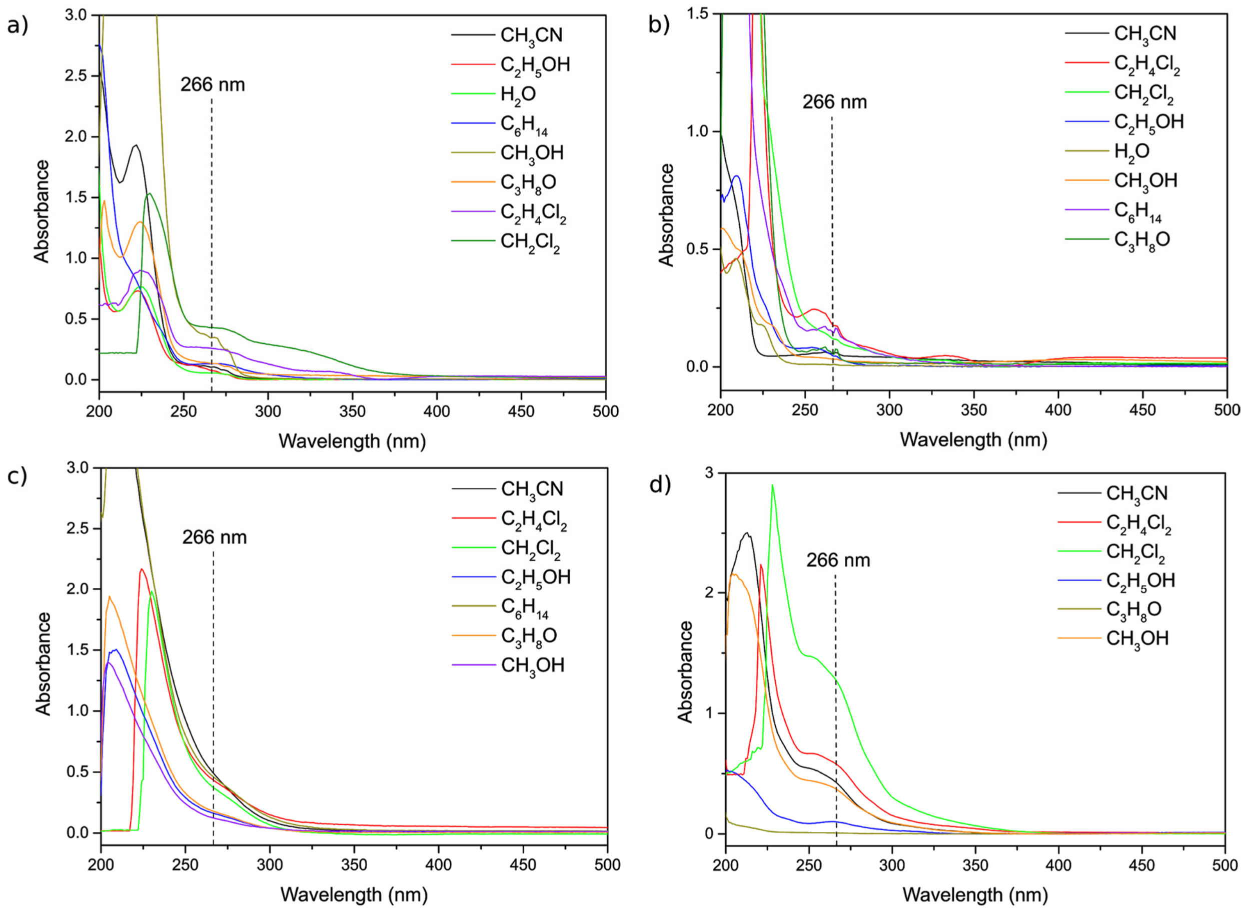


**Figure S2.** Absorbance spectra of precursors in different solvents (**a**) $C_7H_5AgO_2$; (**b**) $C_2AgF_6NO_4S_2$; (**c**) $O[Si(CH_3)_2CH{=}CH_2]_2Pt$; (**d**) $C_8H_{12}Cl_2Pt$.

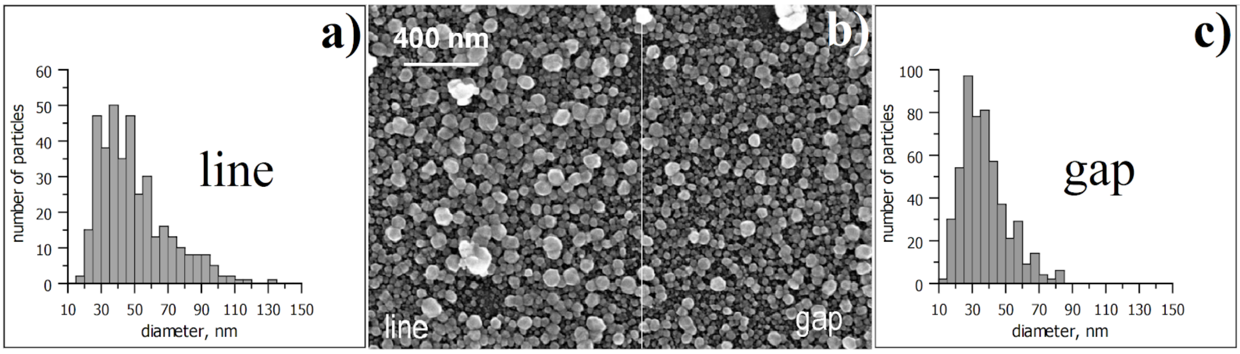


**Figure S3.** (**a**) Particles size histogram for line area of Ag NPs grating; (**b**) SEM for line and gap areas of Ag NPs grating, laser power of 200 mW, irradiation time 20 min periods of the interference pattern 3⌠m (**c**) Particles size histogram for gap area of Ag NPs grating.
